# No Significant Difference in Depression Rate in Employed and Unemployed in a Pair-Matched Study Design

**DOI:** 10.3389/fpubh.2014.00093

**Published:** 2014-07-24

**Authors:** Adriana Mihai, Alina Ricean, Septimiu Voidazan

**Affiliations:** ^1^Department of Psychiatry, University of Medicine and Pharmacy of Tîrgu Mureş, Tîrgu Mureş, Romania; ^2^Child Psychiatry Clinic, Cluj Napoca, Romania; ^3^Department of Epidemiology, University of Medicine and Pharmacy of Tîrgu Mureş, Tîrgu Mureş, Romania

**Keywords:** depression, unemployment, suicide, poverty, risk factors

## Abstract

**Objectives:** The main objective of this study was to evaluate the differences of depression rate in employed and unemployed persons in the period of financial and economic crisis in Romania, in a pair-matched study design.

**Method:** The cross-sectional study uses a pair match design (395 pairs) of two groups of employed and unemployed persons. Other socio-demographic risk factors of depression (gender, age, marital status, residence, ethnicity, educational level, and profession) were controlled. The study was done in a historical period of economic crisis, 2009–2010. For the screening of depression we used the patient health questionnaire-9.

**Results:** There were no statistical differences (*p* = 0.054) between the depression rates in the employed (17.98%) and unemployed (23.80%) samples. The depression rate in both groups was higher in females, age (51–55), marital status (divorced), living in the rural area, with a low level of education and poverty. Suicidal ideas are more frequent in men, employed persons with low level of education and in unemployed persons with medium level of education.

**Conclusion:** The exposure to short term unemployment status was not associated with change in depression rate in the period of financial and economic crisis in Romania, comparing with controls pair-matched. Unemployment status increases the depression rate only in vulnerable groups such as single or divorced women; and suicidal ideas were associated with the unemployment status (longer than 8 months) in men from rural area with medium level of education.

## Introduction

Stable employment, secure incomes, and social capital predict good mental health, while poverty, financial problems, and social deprivation are major socio-economic risk factors for mental health problems (1). On a personal level, the economic crisis could have an impact on many of the social determinants of health, such as income, employment, education, nutrition, corporate practices, and taxation (2). Studies on the unemployment consequences on mental health show that the people who lost their job have a higher risk to develop severe depression (3). Evidence indicates that debt, financial difficulties, and housing payment problems lead to mental health problems (4). Unemployment contributes to depression (5) and suicide, young unemployed people having a higher risk of getting mental health problems than young people who remain employed (2). There are studies which describe poverty as a high risk factor for depression and diabetes (6), with a higher impact on vulnerability groups such as women from rural area (7).

**Figure 1:**
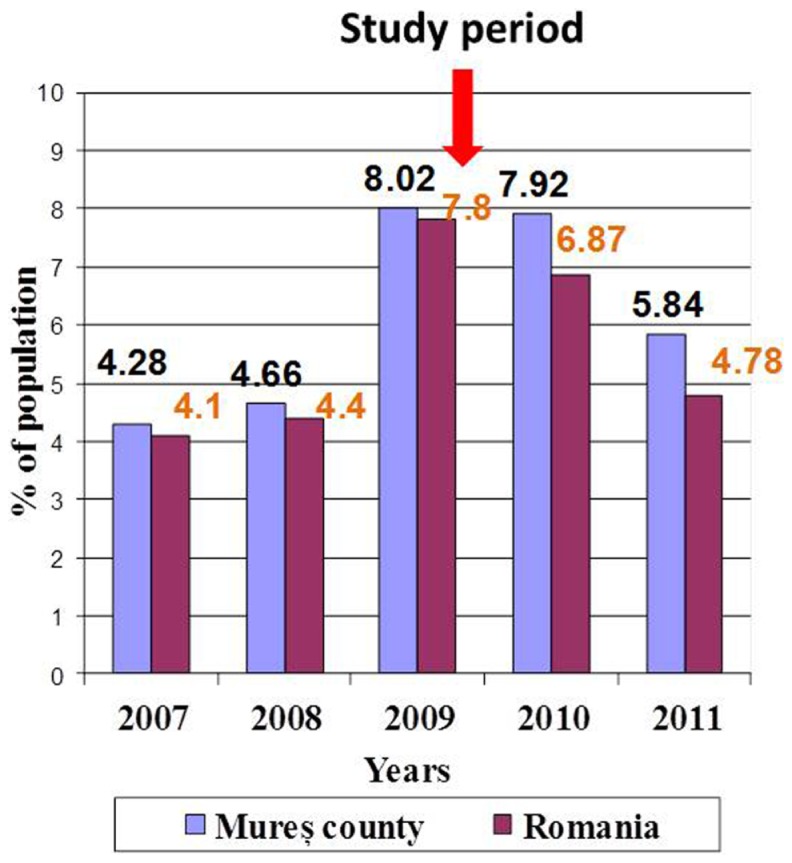
**Unemployment rate during economic crisis 2007–2011**.

The unemployment rate increased abruptly in Romania between 2009 and 2011 because of financial and economic crisis (8, 9). The evolution of unemployment rate as mentioned in official statistics (10) was presented in Figure 1.

In this economical context, the increase of depression rate was expected (11, 12). The question about the impact of unemployment on depression rate arose.

Two hypotheses appeared: (1) the depression rate in employed persons will be lower than in unemployment group during the economic and financial crisis; (2) the depression rate will be not significantly different due to several factors as short term unemployment status (<1 year), attitudes toward poverty and unemployment status, high depression rate in general population.

## Study Objective

The aim of this cross-sectional study was to study the differences of depression rate in employed and unemployed persons in the period of financial and economic crisis in Romania, in a pair-matched study design.

## Materials and Methods

The original idea of this cross-sectional study was to control by a pair match design, all other demographical and social factors, except unemployment status, in order to evaluate the impact of this factor (short term unemployment status) on depression rate in Romanian population during the financial and economic crisis.

We compared two groups: one of unemployed people and second of employed persons 18–65 years old, living in the same administrative county, during the same historical period of economic crisis, 2009–2010.

### Recruitment procedure

The sample size (410 persons) was calculated by statistical methods in relation with the total number of habitants in same period of time and same region (136,000 habitants), the prevalence of depression (15%) in general population (11), the accuracy of estimation 4%, and the significance threshold was *p* = 0.05.

**Figure 2:**
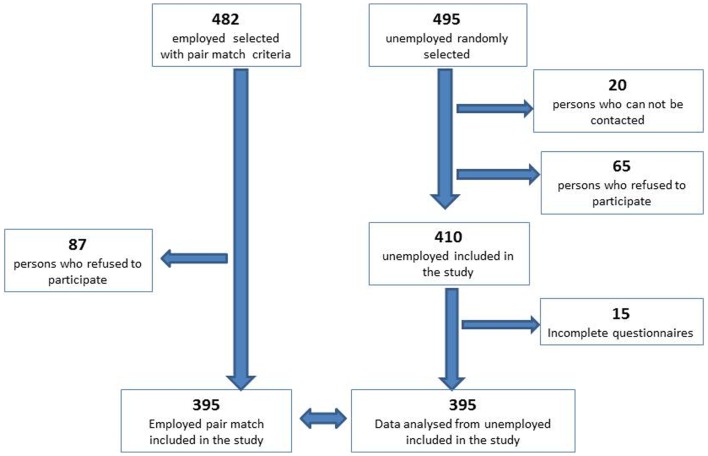
**The flowchart of recruitment**.

We considered short term unemployment status <1 year and we chose this period because the state gives to unemployed person a financial support for maximum 1 year. The unemployed people in order to obtain the state financial support have to visit every month the Workforce Units, on a specific day of the month, in alphabetical order. The randomization consists in selecting every third person, which are on a list at workforce unit. We included all selected unemployed persons who presented themselves to this unit and accepted to answer the study questions until we completed the number (410 persons) decided by the sample size calculation. A number of 20 persons were absent and 65 persons refused to participate in the study. The recruitment period was December 2009, and in 17 days we reached the proposed number, with a response rate of 85.63%. A number of 15 answers were excluded because of incomplete data. A number of 395 persons were included and analyzed (Figure 2).

In the next stage of study, we recruited 395 pair match employed persons, a convenience sample matched for socio-demographic variables (gender, age, marital status, residence, ethnicity, educational level, and profession); in order to eliminate the bias caused by the other socio-demographic criteria, which could be related with the risk of depression. For each person from the unemployed group, we searched a pair match person who works in the same domain of activity as the last place of work of the unemployed person. The process of finding the pair match was performed by a different person than researcher, which applied the questionnaire. Because of different vulnerability to depression described in different ethnic population (13), we included this item in the selection process. In order to obtain this group, we approached 482 persons, with an 82% response rate, 87 refusing to participate. The recruitment period was between December 2009 and June 2010. The interview took place at the participants’ workplace, during the coffee break (Figure 2).

### Inclusion criteria

The inclusion criteria were: employed or unemployed persons living in the evaluated county, age between 18 and 65 years old, and selection according with the recruitment procedure.

### Exclusion criteria

No exclusion criteria other than the refusal to participate in the study. If the person does not speak the official language, Romanian, we use questionnaires translated in the minorities’ languages. If the person could not read, we read them the questions (seven cases).

### Depression screening instrument

A seven page paper containing the study questions was distributed to the participants in the waiting room of the Labor Force Units and waited to be completed on location; we did not accept later answers and considered these situations as refusal to participate in the study. Usually, the unemployed persons have to wait in waiting room more than 30 min, in order to obtain their monthly approval for financial support. The confidentiality of filling the study’s papers was assured.

This study papers contains: (1) informed consent on a separate page and (2) study questionnaire page, which contains: demographical data (age, gender, marital status, residence, ethnicity, educational level, or profession) and self-administrated patient health questionnaire (PHQ)-9 proposed by the World Health Organization for screening depression in primary care, one question about poverty and changes in personal economic situation in the last year.

Patient health questionnaire-9 is translated and validated in Romania and successfully used for screening depression (14). This questionnaire includes each of the nine DSM criteria for depressive episode, being a specific tool for depression screening. The instrument evaluates the symptoms of depressive episode in the last 2 weeks with four options for an answer – ranging from never to nearly every day. A particularly important use of a measure is its responsiveness to changes of condition severity over time. Because of this quality, PHQ-9 is increasingly used as a measure to assess the level of depression severity as well as an outcome tool to determine treatment response. Using the PHQ-9 score we could group responders into four groups: PHQ-9 scores of 0–4 “none or minimal depression,” PHQ-9 scores of 5–9 “mild depression,” PHQ-9 scores of 10–14 “moderate depression,” and PHQ-9 scores of 15–27 “moderately severe or severe depression.” This questionnaire includes one question on suicidal ideas (14–16). Data collected using this questionnaire were self-reported. The questionnaires were administered by a student trained in medicine.

For statistical interpretation of data, we used GraphPad Instat – Chi square test and Fisher’s test. For *p* value <0.05 was considered statistically significant.

### Ethical consideration

Each person had to sign an informed consent in order to be included in the study. These informed consents are kept separately from questionnaire answers in a locked place. For confidentiality reasons, no personal identification data were recorded on the questionnaire. There were no financial consequences from participating in this study. All participants who scored above 5 were informed that they may suffer from depression. For a score between 5 and 15, we recommended to contact their GP and/or mental health services for a medical consultation and a qualified evaluation of depression. For a score above 15, we recommended a qualified specialist consultation and gave them a list of available mental health services in the area and contact details. For those identified with suicidal ideas, we expressed our concern for their life and gave them the list of available mental health services, and a card with a phone contact if they want to make a free appointment to a psychiatrist in a public outpatient clinic for consultation. Collaboration with that setting was previously established. The study received ethical approval from the IRB of the University of Medicine and Pharmacy, Tg Mures, Romania.

## Results

The study was done in the period of economic crisis in Romania, with the higher rate of unemployment in the examined region (Figure 2).

The originality of this study consists in approaching the impact of unemployment on depression with a pair match study design to eliminate the bias caused by other socio-demographic criteria, which could determine depression.

The characteristic of two analyzed samples were presented in Table 1. There are no statistically significant differences in age, gender, marital status, residence, ethnicity, educational level, or profession.

**Table 1:** **Demographical characteristics of both groups: employed and unemployed**.

Demographical characteristics		Unemployed		Employed		*p*	Statistical significance
		*N*	%	*N*	%	
Gender	Male	193	48.86	194	49.11	0.94	NS
	Female	202	51.14	201	50.89	0.94	NS
	Total	395	100	395	100	–	–
Age	<30	122	30.9	133	33.7	0.40	NS
	31–40	105	26.5	106	26.8	0.96	NS
	41–50	118	29.8	110	27.8	0.74	NS
	51–60	50	12.6	46	11.64	0.84	NS
	Total	395	100	395	100	–	–
Marital status	Married	215	54.4	202	51.1	0.49	NS
	Single	125	31.6	148	37.5	0.97	NS
	Divorced	54	13.7	44	11.1	0.69	NS
	Widow	1	0.3	1	0.3	0.99	NS
	Total	395	100	395	100	–	–
Educational level	Low	24	6.1	7	1.8	0.64	NS
	Medium	276	69.8	264	66.8	0.45	NS
	High	95	24.1	124	31.4	0.96	NS
	Total	395	100	395	100	–	–
Domicile	Urban	268	67.8	260	65.8	0.62	NS
	Rural	127	32.2	135	34.2	0.94	NS
	Total	395	100	395	100	–	–
Ethnicity	Romanian	220	55.7	220	55.7	0.99	NS
	Hungarian	173	43.8	173	43.8	0.99	NS
	Romany	2	0.5	2	0.5	0.99	NS
	Total	395	100	395	100	–	–

Thirteen persons (3.29%) under 20 years of age were unemployed just after their high school education without being previously employed, in these cases we considered pair match of professional status the employed persons with the same qualifications training, but we could not find employed persons under 20 years of age, only few years older.

The examined area is characterized by co-habitation of two nationalities – Romanian (50.35%) and Hungarian (46.68%) in almost equal representation, and a Romany minority (2.51%) (12). Due to the known differences in depression vulnerability and the suicidal risk, which is higher in the Hungarian population (13), we included this controlled item in the recruitment process, also we have done statistical analysis on each specific ethnic group, which represents a strength of this study.

**Figure 3:**
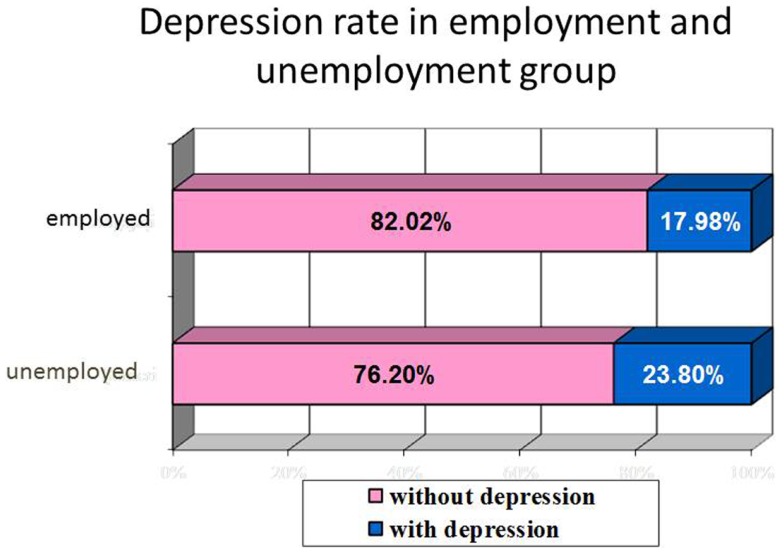
**Correlation between employment status and depression rate**.

We found no statistical differences in depression rate in employed and unemployed group, neither in prevalence nor severity of depression. The depression rate in the employed population was 17.98% comparing with 23.80% in the unemployed population, no statistical differences *p* = 0.054 were obtained (Figure 3).

No statistical differences in depression severity in employed and unemployed group.

The distribution of depression severity was similar in both groups. In the unemployed group 50% (17) have mild depression, 29.79% (18) moderate depression, and 20.21% (19) severe depression and respectively in the employed group 56.34% (20) mild, 25.35% (21) medium, and 18.31% (13) severe depression. The depression rate in the unemployment group slightly increased with the length of the unemployment period, but not statistically significant. The suicidal ideas are more frequent in those with longer period of unemployment.

The depression rate is higher in women than in men in both groups, 2:1 in the employed group, respectively 2.24:1 in unemployed group, but in the unemployed group the women were found with significantly more severe depression than in men (*p* = 0.01).

**Figure 4:**
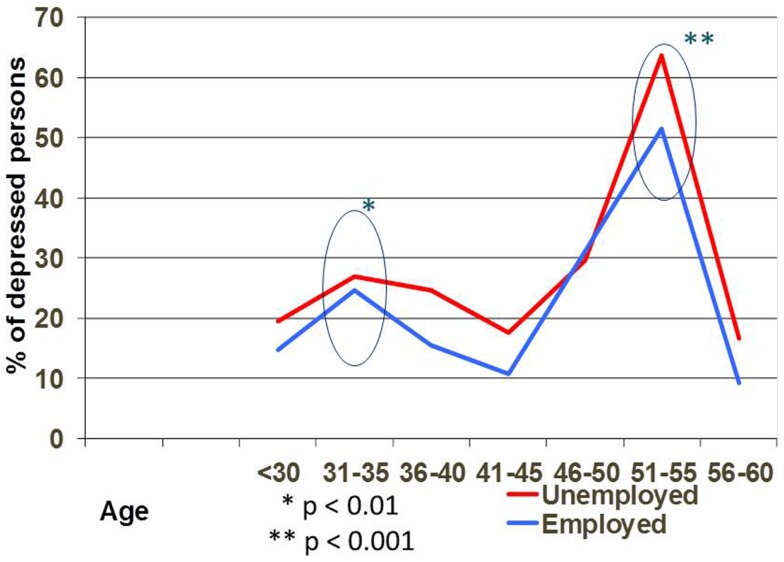
**Depression rate on different age in employed and unemployed persons**.

Analyzing the depression prevalence related to age we observed two peaks: between 31–35 and 51–55 years in both groups, but there are no statistically differences between employed and unemployed groups (Figure 4).

**Figure 5:**
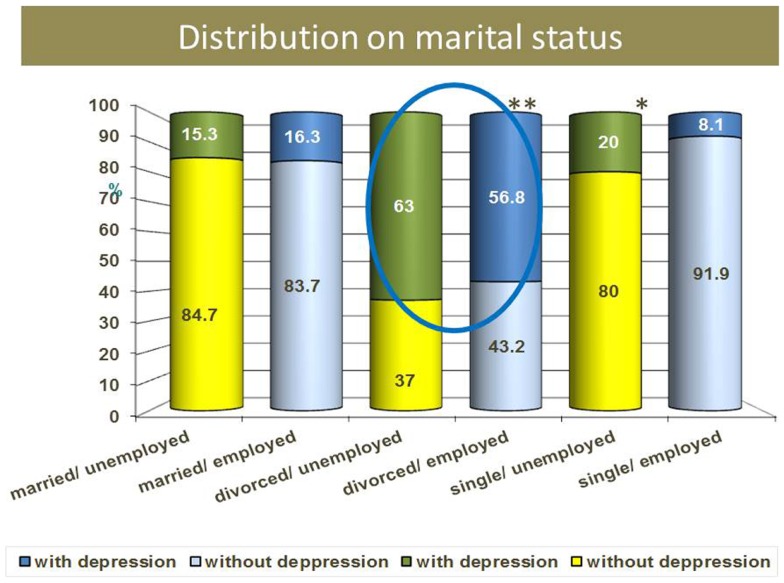
**Depression rate and marital status**.

Comparing the depression rate in both groups related with marital status, we observed that the singles are more at risk to have depression if they are unemployed (*p* = 0.0001), divorced ones and widowers in both genders are more vulnerable to depression no matter if they are employed or not (Figure 5).

**Figure 6:**
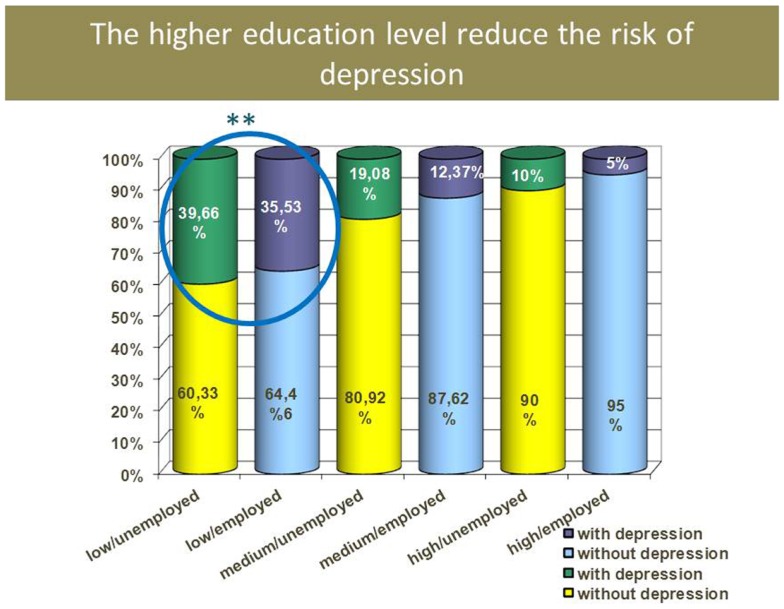
**Depression rate and educational level**.

Low level of education was significantly correlated with higher depression rate in both employed and unemployed groups. With a low level of education, even if the person is employed, the risk to have depression is higher because of the level of poverty, low salary, longer duration of working time, heavy physical work, etc. There is no statistical difference in higher and medium education level regarding the impact on depression rate, no matter if they are employed or not. Five percent of persons who have high level of education and employed are depressed (Figure 6).

Depression rate in rural area (25%) is higher than in urban area (18.7%) not related with employment status, but not statistically significant.

Suicidal risk is significantly higher in employed group of persons with low level of education. 5.31% of persons evaluated reported suicidal ideas, but no statistical differences between employed and unemployed persons, and no statistical difference in relation with ethnicity. Suicidal ideas were more frequent in men, persons from the rural area, and with lower educational level, not related with employment status. The prevalence of suicidal ideation in unemployed group rises proportionally with the duration of unemployment period – from the 21 unemployed persons with autolytic thoughts, 17 (80.90%) had a duration of unemployment period longer than 8 months.

The risk factors for depression in both groups (employed and unemployed) are: female gender, age between 51 and 55 years old, divorced, living in rural area, low level of education and poverty. Suicidal ideas are more frequent in men with low level of education from the employed group, and in men with medium level of education from the unemployed group.

## Discussions

The research on mental health revealed that unemployment, poverty, and loss of family could be related with depression, alcohol use disorders, and suicide (4, 19, 21–23). In our study, the depression rate in the unemployed group was not significantly higher than in the employed group.

Studies performed in the actual financial crisis, as those of Wang et al. (24) and Lee et al. (25), also show a rise in the prevalence depression for the employed people (24, 26). Related factors that could explain the increase in depression rate in employed persons during the economic crisis could be higher burden both at the work place because of less employees, and at home due to less revenue and increased prices. In our study, the rate of depression in employed group was 17.98%. In the employed lot, 23% (27) have developed depression from those who reported that income decreased in last year (196), and only 13.06% (24) from those who did not experience financial loss in last year (199) – statistically significant association (*p* < 0.01) between depression rate and change in the financial status. These results are consistent with other longitudinal studies, which showed that the financial deficit had a negative effect, while the reduction of the financial difficulties had a positive effect on the depression score (25, 28, 29).

Other explanation of higher depression rate in employed population could be the legacy of the communist period associated with challenges of new economic system: poverty; bureaucratic system; political, economic, and financial instability; adjustment difficulties to new capitalist system. The depression rate, defined as a point prevalence of depression in the last 2 weeks, for general population seems to be similar with other Central – Eastern European post-communist countries in a study done prior the economic crisis (30). We found in Romania an average of 20.89% depression rate in both groups employed and unemployed, but calculated for the population aged between 45 and 65 years old, as studied population in the Bobak study this value became 32.3% for Romania similar with 33.5% in Russia, 25.5% in Poland, and 26.5% in Czech Republic (30).

Our results are also consistent with the Weich and Lewis (31) study, which did not find a correlation between the job loss and the onset of depression, but only a correlation between unemployment and the duration of the depressive episode (18).

Our study suggests that a length of unemployment longer than 8 months, period probably related with using resources and increase poverty, was correlated with the higher risk of depression but not significant statistically.

Previous proofs show that the depression frequency rises continuously in the first 6 months of unemployment, and then reaches a plateau and decreases with employment (3). Link and Phelan (32) proposed the low socio-economic status as a “fundamental cause” of the disease (31). Other possible mechanisms of depression may include: insufficient social support, low control on the own environment, and the not favorable comparison with other people (32). These findings support the idea than poverty level could be more related with an increase in the prevalence of depression, rather than unemployment status.

The rate of those living below the level of poverty line during the financial and economic crisis period increased. In our study, the income of half of the employed people decreased because of the economic crisis, and one-third “fall” under the poverty line. The poverty line is the minimum level of income deemed adequate in a particular country (33). The poverty line is established by the national authorities who estimate the cost of living. The standards are different from country to country. In Romania, 13.8% of the population in 2006 and 21.1% in 2010 were below the poverty line (34).

Shapinakis’s study showed that the subjective perception of financial difficulties at the initial moment was associated independently with depression (35). The idea that depression is caused more by the subjective perception of possible consequences of financial difficulties than financial difficulties themselves, sustain our second hypothesis. The hypothesis was that the previous history of poverty during the communist period could protect Romanians against depression; they will not have a catastrophic perspective of the future and will not be scared by some financial difficulties. In order to verify this hypothesis we need, besides the objective quantification of the socio-economical position, more psychological investigation on attitude and values, which were not done in this study.

Analyzing the depression prevalence in relation with age we observed two peaks: between 31–35 and 51–55 years in the both groups, but there are no statistical differences between employed and unemployed groups. The level of depression severity was significantly higher in the 51–55 years old group (*p* < 0.001), not related with employment status. This age could be more vulnerable because of other factors such as adult children living with their parents, change in health status, and for those unemployed the difficulties of finding a new job due to employers’ preference for younger candidates, etc. Other studies demonstrate that social stress, health status, and psychosocial resources influence the strength and shape of the age-depression relationship (32, 36).

In the rural area, frequently only one member family is employed and the others work on their own farm. The loss of employment of the key member could significantly impair the family life, which could explain the higher level of depression in this group.

Concerning the suicidal ideas, the literature shows that, especially men are at increased risk of mental health problems and death due to suicide or alcohol use during times of economic adversity (37, 38). Suicide is more common in areas of high socio-economic deprivation, social fragmentation, and unemployment (20, 39–41). An increased income inequality has been linked to an increase in suicide rates (42).

In our study, 5.31% of the evaluated persons reported suicidal ideas, but no statistical differences between employed and unemployed persons. There was no statistical difference in relation with ethnicity, we expected to find a higher frequency in the Hungarian population as showed in other studies, but it was not confirmed. Suicidal ideas were more frequent in men, persons from rural area, with lower educational level, and were not related to employment status. Suicidal risk is significantly higher in employed group of persons with low level of education. The prevalence of suicidal ideation in unemployed group rises proportionally with the duration of the unemployment period – from the 21 unemployed persons with autolytic thoughts, 17 (80.90%) had a duration of unemployment period longer than 8 months. The low number for those who reported suicidal ideas made it difficult to evaluate the impact of other covariates.

Suicides are significantly more likely to be unmarried, unemployed, and non-skilled than the members of the control group. Men and women who are unmarried (never married, divorced, or widowed) have higher suicide rates than people who are married, marital status having a greater protective effect on men than on women (43). Overall, men have the highest rate of completed suicides, whereas women have the highest lifetime rate of suicide attempts (44). Having children appears to attenuate risk (27) and number of children has been negatively associated with suicide risk in women (45). Suicide attempts and completed suicides are more prevalent in unemployed compared with employed populations (46, 47). Unemployment has been found to place women at greater risk of suicide relative to males (45).

### Study strengths

The high quality of the used instrument, PHQ-9, which was translated and validated in Romanian and Hungarian increases the power of obtained results (14, 16). The sample size for this type of study design is well represented.

The peculiarity of this study, compared to other studies on the same topic, is that it was done on a population, which until 1989 was under the communist coordination and lived in a known level of poverty and deprivation: food, water, and electricity was rationalized, the population was employed but the salary was very low. The impact of this specific historic and cultural background on the level of poverty tolerance could be reflected as a protective factor in an economic crisis.

### Limitations of the study

Several limitations of the study need to be considered.

#### Limitations related with the used instrument

Depressive symptoms and other variables were self-reported. Some of the covariates are subjective, such as the rating of the changes in personal economic status in last year. For depression rate, we used a screening instrument and not a precise diagnosis. PHQ-9 is translated and validated both in Romanian and Hungarian, and has a good validity and reliability. Non-response bias should also be considered, as in general, people who participate in health surveys are healthier than those who do not (30). We obtained a good response rate similar in both groups of employed and unemployed persons (>80%), but the levels of depressive symptoms in our study are probably underestimated. Some important risk or protective factors were not evaluated: personal history of mental health problems, previous suicidal attempts, unstable personality disorder, alcohol use, religious status, number of children, family income structure, etc.

#### Limitations related with the study period

The study was done in December 2010, the month of Christmas and winter holidays. The Romanians show a high respect for these events and frequently they spend more than they can afford in order to organize these family events. This is an opportunity of joy to be together with the entire family and hope that together they will pass through difficulties, which could reduce the reported depression rate, but in the economic crisis this period could also determine an increase of self-reported depression due to the lack of financial resources for the minimal needs in respecting tradition.

#### Limitations related with the recruitment procedure

The economic crisis affect the majority of working fields, but it does not lead to an increase of unemployment rate in all of them. The pair match style involved the evaluation of the same economic domain where the unemployment rate existed. The domains where the crisis lead not to job loss, but only to the reduction of salary, such as medical or education fields, were not represented in the study. The employed persons included were from the same affected economic field like those unemployed, with a higher risk to lose their jobs, because of this reason they could have higher depression rate than in general population. The recruitment procedure excludes those unemployed persons who do not receive financial support from the state. We could assume that the impact of unemployment could appear later than 12 months period of state financial support.

## Conclusion

The exposure to short term unemployment status in the period of financial and economic crisis in Romania was not associated with higher depression rate than comparing with controls employed pair-matched. There were no statistical differences in depression rate or severity of depression in employed and unemployed group. The risk factors for depression in both groups (employed and unemployed) are: female gender, age between 51 and 55 years old, divorced, living in rural area, low level of education and poverty.

Unemployment status increases the depression rate only in vulnerable groups as single or divorced women; and suicidal idea were associated with unemployment status in men from rural area with medium level of education and with duration of unemployment longer than 8 months.

## Conflict of Interest Statement

The authors declare that the research was conducted in the absence of any commercial or financial relationships that could be construed as a potential conflict of interest.
